# Kinetics of Cu, Pb and Zn removal during soil flushing with washing agents derived from sewage sludge

**DOI:** 10.1038/s41598-021-89458-z

**Published:** 2021-05-12

**Authors:** Barbara Klik, Zygmunt M. Gusiatin, Dorota Kulikowska

**Affiliations:** Department of Environmental Biotechnology, Faculty of Geoengineering, University of Warmia and Mazury in Olsztyn, 10-719 Olsztyn, Poland

**Keywords:** Environmental chemistry, Environmental impact, Environmental sciences

## Abstract

This paper presents the first tests of Cu (7875 mg/kg), Pb (1414 mg/kg) and Zn (566 mg/kg) removal from contaminated soil with sewage-sludge–derived washing agents (SS_WAs) (dissolved organic matter, DOM; soluble humic-like substances, HLS; soluble humic substances, SHS) and Na_2_EDTA (as a standard benchmark) in column experiments. Flow rates of 0.5 ml/min and 1 ml/min were used. Using a 1. order kinetic model, the kinetic constant (*k*), the maximum concentrations of each metal removed (*C*_max_), and the initial rates of metal removal (*r*) were established. At both flow rates, stable flow velocity was maintained for approximately eight pore volumes, for flushing times of 8 h (1.0 ml/min) and 16 h (0.5 ml/min). Although the flow rate did not influence *k,* it influenced *C*_max_: at 1 ml/min, *C*_max_ values were higher than at 0.5 ml/min. For Cu and Zn, but not Pb,* k* was about twofold higher with Na_2_EDTA than with SS_WAs. Although Na_2_EDTA gave the highest *k*_Cu_, *C*_max,Cu_ was highest with DOM (Na_2_EDTA, 66%; DOM 73%). For Pb removal, HLS was the most effective SS_WA (77%; Na_2_EDTA was 80% effective). *k*_Zn_ was about twofold higher with Na_2_EDTA than with SS_WAs. *C*_max,Zn_ was highest with HLS. The quick mobilization of Cu, Pb and Zn with most of the WAs corresponded to efficient metal removal from the exchangeable (F1) fraction.

## Introduction

Due to their persistence, bioaccumulation and toxicity, heavy metals (HMs) should be eliminated from soil, or their negative impact on the environment should be diminished. HMs are as commonly found in the environment as other contaminants like mineral oils or aromatic hydrocarbons. In many areas associated mainly with present and former industrial activities, the concentrations of HMs are several or even several dozen times over the permissible values^1–7^.

Among the many remediation methods, soil washing and soil flushing enable the permanent removal of HMs from soil and therefore are preferable to immobilization of contaminants. Up to now, most studies have reported results from batch soil-washing experiments, which have aimed to optimize this process with the use of various soils and HM contamination levels, and various washing agents under different soil-washing operational conditions^7–12^. In contrast to batch soil-washing, soil-flushing methods for HM removal are still under development^13^, although this is generally considered a mature technology because it has been used for decades in oil field applications to enhance oil recovery^14^. In soil flushing, contaminants are removed from soil by passing the washing agent through the soil. Then, the spent washing agent is recovered, reused, and eventually treated and disposed of. This technology is applicable to homogenous, coarse-textured soils with high permeability, and its main advantages are the elimination of the need to excavate, handle, and transport large quantities of contaminated soil, and its relatively small surface footprint, which allows it to be used in space-limited areas^15^.

So far, the same types of solutions have been tested for soil flushing as for batch soil-washing: acidic, basic, chelating, reducing and surface-active. Among these, Na_2_EDTA, mineral acids and organic acids have been most intensely studied and have proven to be the most effective^16–21^. However, the literature clearly indicates that, in soil-flushing research, authors have focused mainly on process effectiveness. For example, Yang et al.^19^ used bench-scale column washing to examine the efficiency of removal of DTPA-extractable HMs (Cd, 12.82 mg/kg; Pb, 105.38 mg/kg) using different doses of HCl and Na_2_EDTA in a contaminated calcareous soil. In sequential use of the optimal doses of HCl and Na_2_EDTA, DTPA-extractable Cd and Pb were removed with efficiencies of 87.3% and 73.2%, respectively. Compared to the use of a single washing agent, the efficiencies of removal of DTPA-extractable Cd and Pb were increased by more than 8.3% and 27.92%, respectively, by sequential washing with HCl followed by Na_2_EDTA.

Even in the most recent work on soil flushing, research has focused mainly on the effectiveness of pollutant removal, most often using a variety of conventional washing agents (WAs)^16,17,20,21^. HM removal in soil flushing with next-generation WAs, especially those recovered from wastes, has rarely been reported. Moreover, there is gap in the knowledge about the relationship between next generation WAs flow rate and the efficiency of HM removal during soil flushing, although it is known that flow intensity affects the efficiency of organic pollutant removal^22^. The effectiveness of soil flushing can be associated with the kinetics of desorption or dissolution of HMs from soil constituents^23^. Thus, it is crucial to better understand the performance of flushing agents in removal of HMs from soil, especially with regard to next-generation agents, i.e. those extracted from wastes.

In view of the above considerations, this study compared for the first time the efficiency of next-generation, sewage-sludge–derived WAs (SS_WAs) (dissolved organic matter, DOM; soluble humic-like substances, HLS; soluble humic substances, SHS) and Na_2_EDTA (as a standard benchmark) for Cu, Pb and Zn removal in a column experiment that simulated soil flushing. The primary objectives were as follows: (1) to estimate the efficiency of HM removal with the tested WAs, (2) to examine the impact of WA flow rate on total HM removal and chemical fractions of HMs, and (3) to estimate the kinetic constants of the process.

## Materials and methods

### Soil

Surface soil samples (0–30 cm) were collected from agriculture area in Troszkowo (54°3′18″ N, 20°53′28″ E, Warmia and Mazury Province, Poland). In total, 10 kg of soil (through mixing of 5 sub-samples) was collected from area of 5 m^2^. In laboratory, the soil was air-dried for two weeks and ground to pass through a 2 mm sieve. The homogenous soil was then spiked with a mixture of Cu, Pb and Zn. For spiking, nitrate salts of HMs i.e. Cu(NO_3_)_2_, Pb(NO_3_)_2_ and Zn(NO_3_)_2_ (Sigma-Aldrich) were used. The salts were dissolved in distilled water to achieve concentrations, 8000 mg/L of Cu, 1500 mg/L of Pb and 500 mg/L of Zn. The solutions were added directly to 1 kg of soil. The soil was mixed for 24 h on a horizontal mechanical shaker (Laboshake Gerhardt). Next, the soil was incubated at room temperature (23 ± 2 °C) for three months with gentle, frequent mixing. The soil characterized with the following characteristics: 56 ± 1.6% sand, 39 ± 0.16% silt, 5 ± 0.21% clay, pH 6.4 ± 0.1, 3.4 ± 0.1% organic matter, 17.2 ± 1.6 cmol/kg cation exchange capacity, 7874.5 ± 23.1 mg/kg total Cu, 1414.3 ± 11.6 mg/kg total Pb, 566.1 ± 4.2 mg/kg total Zn. The concentrations of HMs in the spiked soil corresponded to the concentrations in soils in close proximity to Legnica smelter (Poland)^7^. Detailed soil characteristics are given in Klik et al.^24^.

### Washing agents

Three washing agents (WAs) derived from municipal sewage sludge (SS_WAs) were used for HM removal in column soil flushing:

- Dissolved organic matter (DOM) washed from sewage sludge with distilled water,
- Humic like substances (HLS) extracted from sewage sludge with 0.1 M NaOH,
- Soluble humic substances (SHS) extracted from sewage sludge with 0.1 M NaOH after sludge washing with distilled water to eliminate soluble non-humic substances (e.g. sugars and proteins) and sludge defatted with a chloroform:methanol mixture^25^.

The conditions for extraction of SS___WAs from municipal sewage sludge are summarized in Table 1. As a benchmark for comparison, commercially available Na_2_EDTA (Sigma-Aldrich) was used. Selected characteristics of SS_WAs and Na_2_EDTA were given in Table 2^24^. Based on previous research, for column flushing experiments, the SS_WAs were used at concentration of 5 g C/L and at pH 4.0, and Na_2_EDTA at concentration of 0.64 g C/L and at pH 4.0^24,26^.

**Table 1:** Conditions for extraction of SS_WAs.

Parameter	Type of WA		
	DOM	HLS	SHS
Solution used for extraction	Distilled water	0.1 M NaOH	0.1 M NaOH*
m/V ratio** (g/ml)	1/10	1/10	1/8
Extraction time (h)	1	6	6
Shaking speed (rpm)	150	150	150
Centrifuging (speed/time)	10,000 rpm/10 min		
Filtration	vacuum filtration, 0.45 μm filter		

*Before the proper extraction of SHS, dissolved substances, waxes, bitumens were removed; **m/V is the ratio between sludge mass (g) and volume of extractant (ml).

**Table 2:** Selected properties of WAs used for soil flushing (mean value, n = 3, ± standard deviation).

Characteristic	DOM	HLS	SHS	Na_2_EDTA
pH	6.9 (± 0.08)	11.7 (± 0.13)	12.3 (± 0.11)	4.6 (± 0.09)
Concentration (g/L)	6.8* (± 0.24)	9.7* (± 0.42)	5.0* (± 0.19)	0.64 (± 0.19)
NH_4_^+^ (mg/L)	331.2 (± 19.8)	143. 0 (± 11.1)	134.4 (± 18.1)	1.6 (± 0.9)
PO_4_^3−^ (mg/L)	2850.4 (± 162)	2496.8 (± 141)	1015.2 (± 73)	1.4 (± 0.6)
Mg^2+^ (mg/L)	15.0 (± 2.4)	1.7 (± 0.26)	1.4 (± 0.11)	0.014 (± 0.006)
Ca^2+^ (mg/L)	0.8 (± 0.09)	11.5 (± 2.2)	17.8 (± 3.9)	0.11 (± 0.03)
Cu^2+^ (mg/L)	1.1 (± 0.05)	2.3 (± 0.12)	4.7 (± 0.19)	0.7 (± 0.19)
Zn^2+^ (mg/L)	0.7 (± 0.08)	1.3 (± 0.12)	3.6 (± 0.32)	1.6 (± 0.32)
Pb^2+^ (mg/L)	0.0	0.0	0.0	0.0
Cd^2+^ (mg/L)	0.0	0.0	0.0	0.0

*original DOM, HLS and SHS concentration (expressed as g of dissolved organic carbon per L) extracted from sewage sludge under conditions given in Table 1.

### Soil flushing

**Figure 1:**
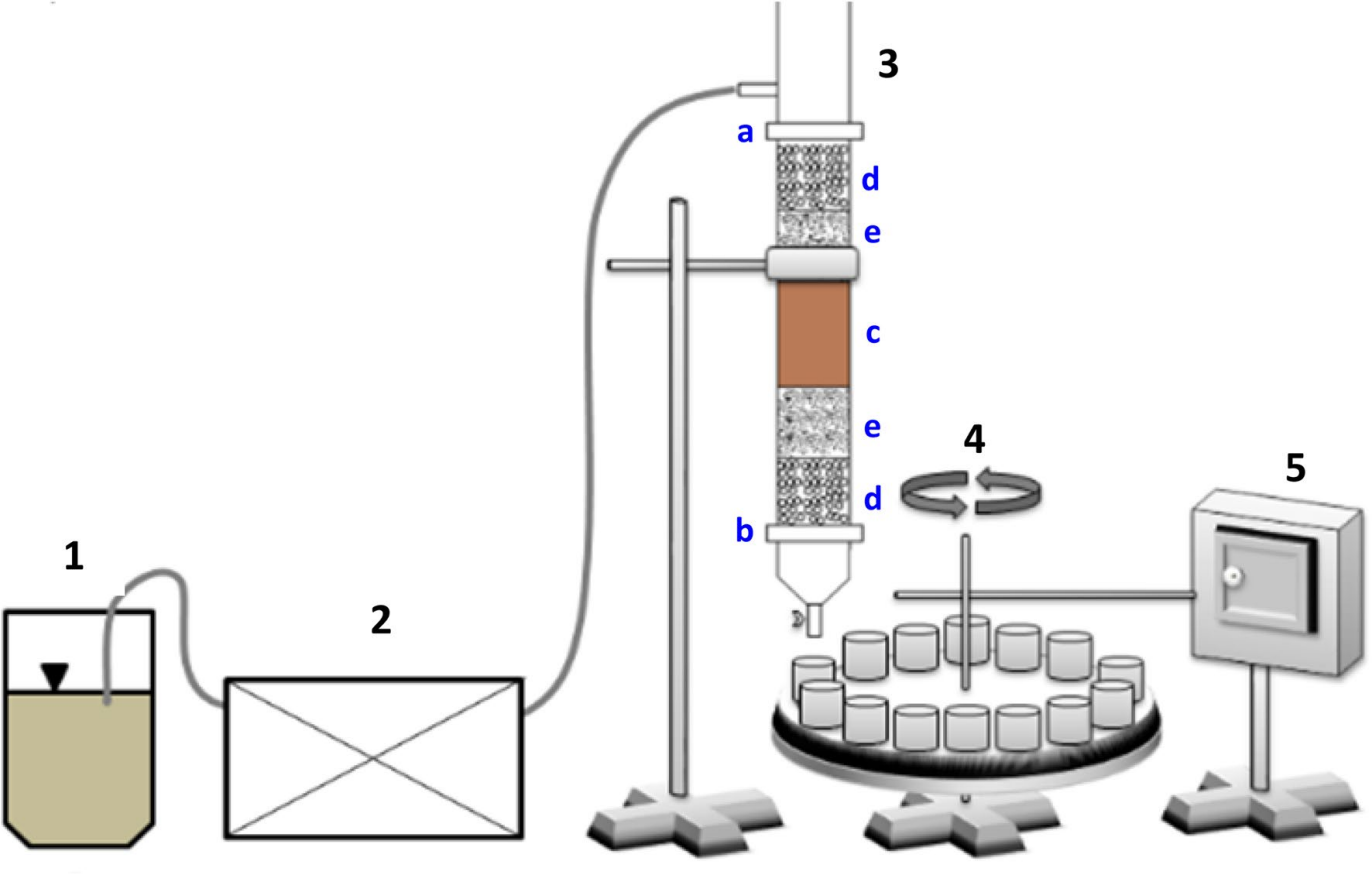
Set-up for metal removal in soil flushing: container with flushing agent (1), peristaltic pump (2), column reactor (3): supporting sieve (**a**), closing sieve (**b**), soil (**c**), ϕ 2–4 mm gravel layer (**d**), ϕ 1–2 mm gravel layer (**e**), automatic sampling device (4), control cabinet (5).

The experiments were performed in a column reactor equipped with additional devices presented in Fig. 1.

The column reactor was made of plexi with a 30-mm internal diameter and a 300 mm length. The column had two sieves (a supporting sieve at the top of column and a closing sieve at the bottom with a nylon mesh) enabling proper maintenance of soil and gravel layers and to ensure the leachate were free of turbidity during flushing. The column was packed with two layers of rinsed gravel (φ 2–4 mm and 1–2 mm) (total mass of 200 g) and a layer of contaminated soil (total mass of 50 g). The mass of soil in the reactor was chosen experimentally to obtain a suitable flow rate with tested WAs through the reactor. The bulk density of the soil was 1.23 g/ml with a porosity on the level of 54%. The pore volume of the packed column reactor was 56.8 ml.

The individual WAs were pumped into column reactor with a peristaltic pump (LeadFluid BT600S) at two flow rates, i.e. 0.5 ml/min and 1.0 ml/min. The total time of soil flushing with individual WAs was 24 h. The flushing solution was pumped into the column from the top layer, while the leachate was recovered from the bottom. This flushing method, in contrast to soil flushing from the bottom to the top, simulates better field application where the soil is subjected to a constant water head or other solution application from the top^27^.

Before soil flushing with individual WAs, distilled water was pumped into the reactor for 1 h to saturate the soil. After this pre-conditioning, WAs were continuously applied. The first volume of water leachate was withdrawn.

**Figure 2:**
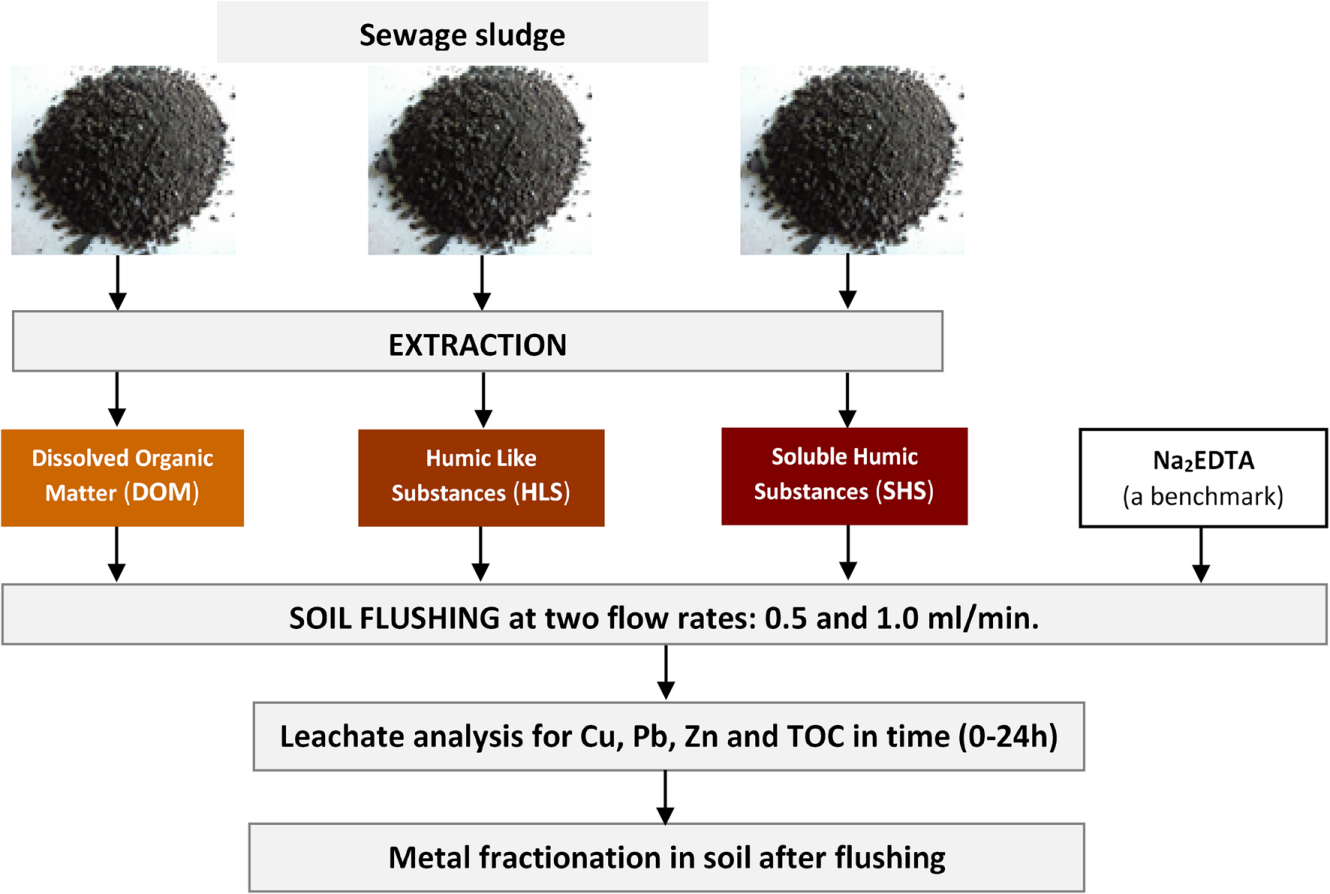
Research scheme on soil flushing with WAs.

The leachate from the column were collected from the bottom of the reactor by the automatic sampling device (in form of rotor with 36 leachate containers). The advantage of using this automatic devise was the possibility of leachate collection at every hour. All column experiments were conducted at room temperature, under laboratory conditions. The average total leachate volume for each WA was 654 ml (0.5 ml/min) and 1210 ml (1.0 ml/min). The research scheme on soil flushing with tested WAs is shown in Fig. 2. The soil flushing with each WA was evaluated in duplicate columns.

### Analyses of column leachate and flushed soil

The leachate from column reactor were collected regularly for 24 h at 1-h interval. In the raw leachate samples pH was measured using a pH-meter (HI 221). Then samples were filtered using 0.45 µm nitrocellulose membrane filters and stored at 4 °C prior to chemical analyses. Concentration of dissolved HMs (Cu, Pb, Zn) were measured in samples acidified with 65% HNO_3_ using flame atomic absorption spectrometer, FAAS (AA280FS, Varian). Detection limits for Cu, Pb and Zn were 0.004 mg/L for Cu, 0.003 mg/L for Zn and 0.01 mg/L for Pb. The leachate samples after 0, 1, 2, 5, 10 and 24 h were also subjected to analysis of total dissolved organic carbon (TOC) with Shimadzu Liquid TOC-VCSN analyzer (calculated by subtracting the inorganic carbon from the total carbon).

The soil after flushing was air dried, crushed and sieved through a 1 mm sieve for the determination of chemical fractions of HMs. Fractions of Cu, Pb and Zn were determined using a modified BCR (Community Bureau of Reference) sequential extraction procedure. Four operationally defined fractions were determined: exchangeable and acid soluble (F1), reducible (F2), oxidizable (F3) and residual (F4). The details on the BCR procedure were given in Pueyo et al.^28^. For HM measurement quality assurance, the certified reference material (BCR-142R) was used. For calibration curves, HM standards for AAS (Fluka Analytical) of concentration of 1000 mg/L each one were used. Repeatability of FAAS analyses was confirmed by triplicate reading of HM concentrations in samples with relative standard deviation (RSD) < 2%.

### Results elaboration

The removed HM concentrations were plotted against the soil flushing time to construct the mobilization curves of HMs. Cumulative HM removal from the soil column with individual WAs was calculated as the sum of HM removed at each hour of soil flushing. Based on TOC concentration in original WAs and leachate from column, the sorption of WAs was evaluated. Total HM removal during soil flushing was conjuncted with analysis of the efficiency of HM removal from their individual fractions (F1–F4).

Based on cumulative HM removal, kinetic constants were calculated, according the 1. order kinetic formula:

$$C = C_{{{\text{max}}}} \cdot \left( {1 - e^{{ - k \cdot t}} } \right)$$

where: *C*_max_ is the maximum concentration of the individual HMs (Cu, Pb, Zn) removed from washed soil (mg/kg); *k* is the kinetic constant of the individual HM removal (h^−1^); *t* is the soil flushing time (h). On the basis of *C*_max_ and *k*, initial rate of individual HM removal *r* (mg/kg h) was calculated.

To ensure the accuracy, reliability, and reproducibility the soil flushing experiments were performed in duplicate. The data in figures are the average of duplicates, and the error bars indicate the standard deviation of the repetitions.

## Results and discussion

### Hydraulic conditions and sorption of WAs during soil flushing

**Figure 3:**
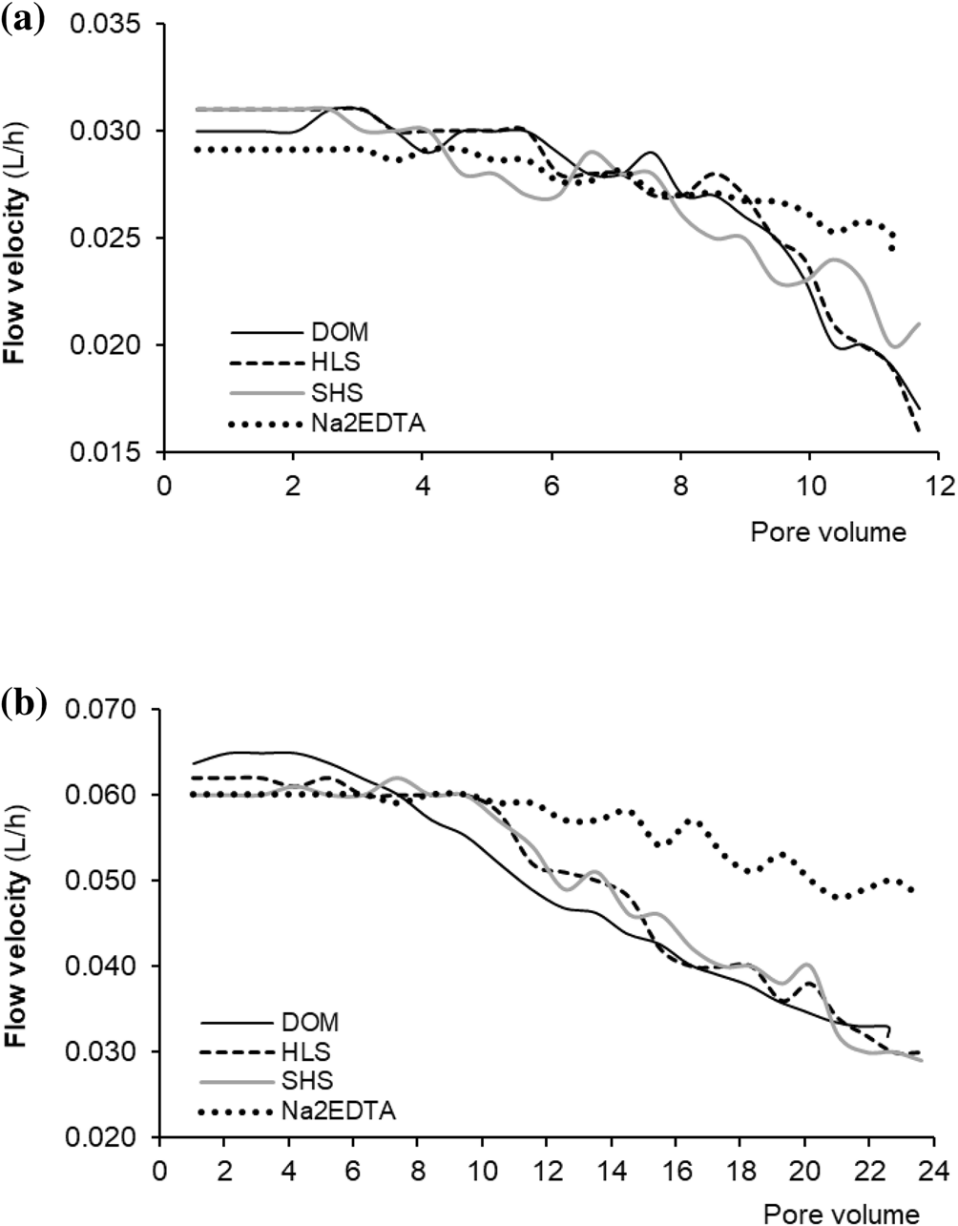
Flow velocity and pore volume during soil flushing with DOM, HLS, SHS and Na_2_EDTA.

The hydraulic conditions in the soil flushing reactor are presented in Fig. 3. These conditions depended on both the type of WA and the flow rate. As expected, the observed flow velocity was twofold higher at 1.0 ml/min than at 0.5 ml/min. As a result, the soil in the column reactor was flushed with more pore volumes during a shorter flushing time at the higher flow rate than at the lower rate.

With all WAs at both flow rates, stable flow velocity was maintained for approximately eight pore volumes, which corresponded to flushing times of 8 h at 1.0 ml/min and 16 h at 0.5 ml/min. With longer flushing times and larger pore volumes, hydraulic conditions became unstable. This destabilization was more noticeable at the higher flow rate, particularly with SS_WAs (Fig. 3). Changes in hydraulic conditions are typical during soil flushing. At higher chelating-agent concentrations, the decrease in flow velocity was greater with EDDS than with EDTA^16^. In the present study, the concentration of SS_WAs was higher than that of EDTA. Higher washing-agent concentrations can result in plugging due to dispersion of fine soil fractions^29,30^.

During soil flushing, the pH in the column leachate continuously decreased. At the beginning it ranged from 4.9 to 5.1 at 0.5 ml/min and from 4.6 to 4.9 at 1.0 ml/min (full data not shown). At the end of soil flushing, the pH had decreased to 4.1 to 4.3 at both flow rates. Yun et al.^30^ demonstrated that a decrease in the pH of leachate to a value ranging from 3 to 5 during soil flushing with citric acid could increase the electrostatic forces between soil particles and cause swelling of fine particles, resulting in decreased soil permeability to the flushing agent.

**Figure 4:**
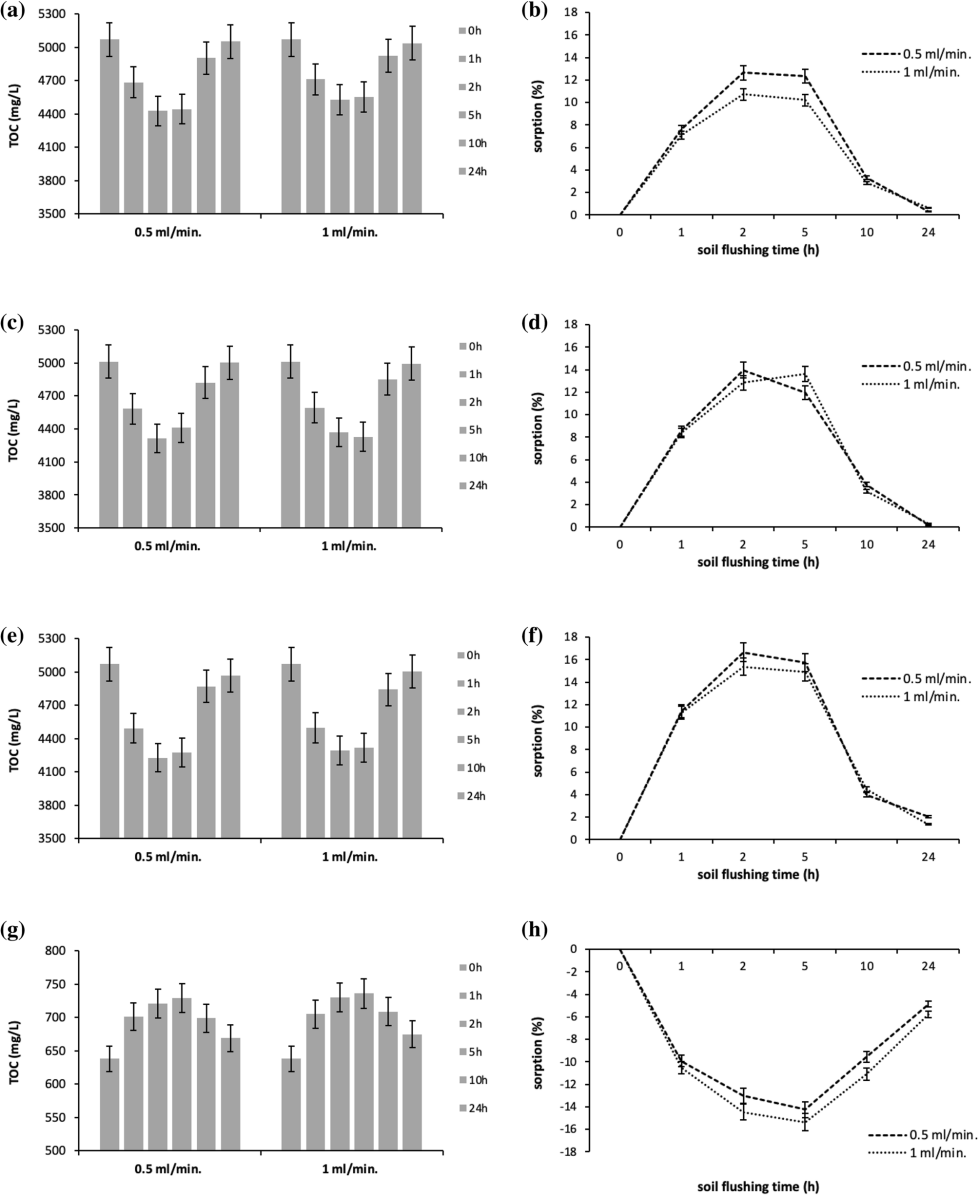
Changes in the concentration of WAs (as TOC) in leachate collected, and the sorption of WAs at specific flushing time (0–24 h) at two flow rates: (**a**,**b**) DOM, (**c**,**d**) HLS, (**e**,**f**) SHS, (**g**,**h**) Na_2_EDTA. Data are expressed as mean values (n = 2) ± standard deviation error bars.

**Figure 5:**
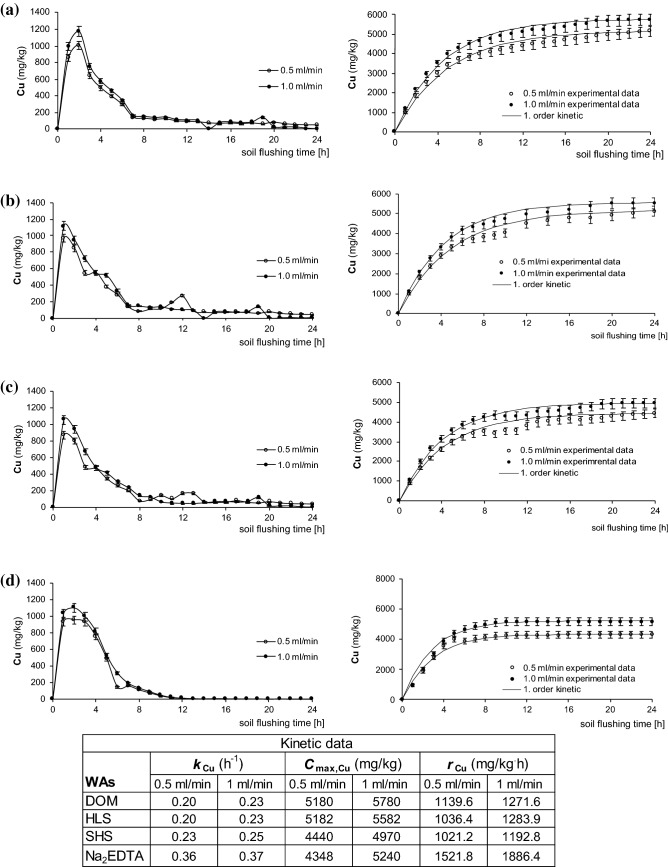
Cu mobilization curves and its cumulative removal during soil flushing at two flow rates with DOM (**a**), HLS (**b**), SHS (**c**), Na_2_EDTA (**d**) with kinetic constants. Data are expressed as mean values (n = 2) ± standard deviation error bars.

**Figure 6:**
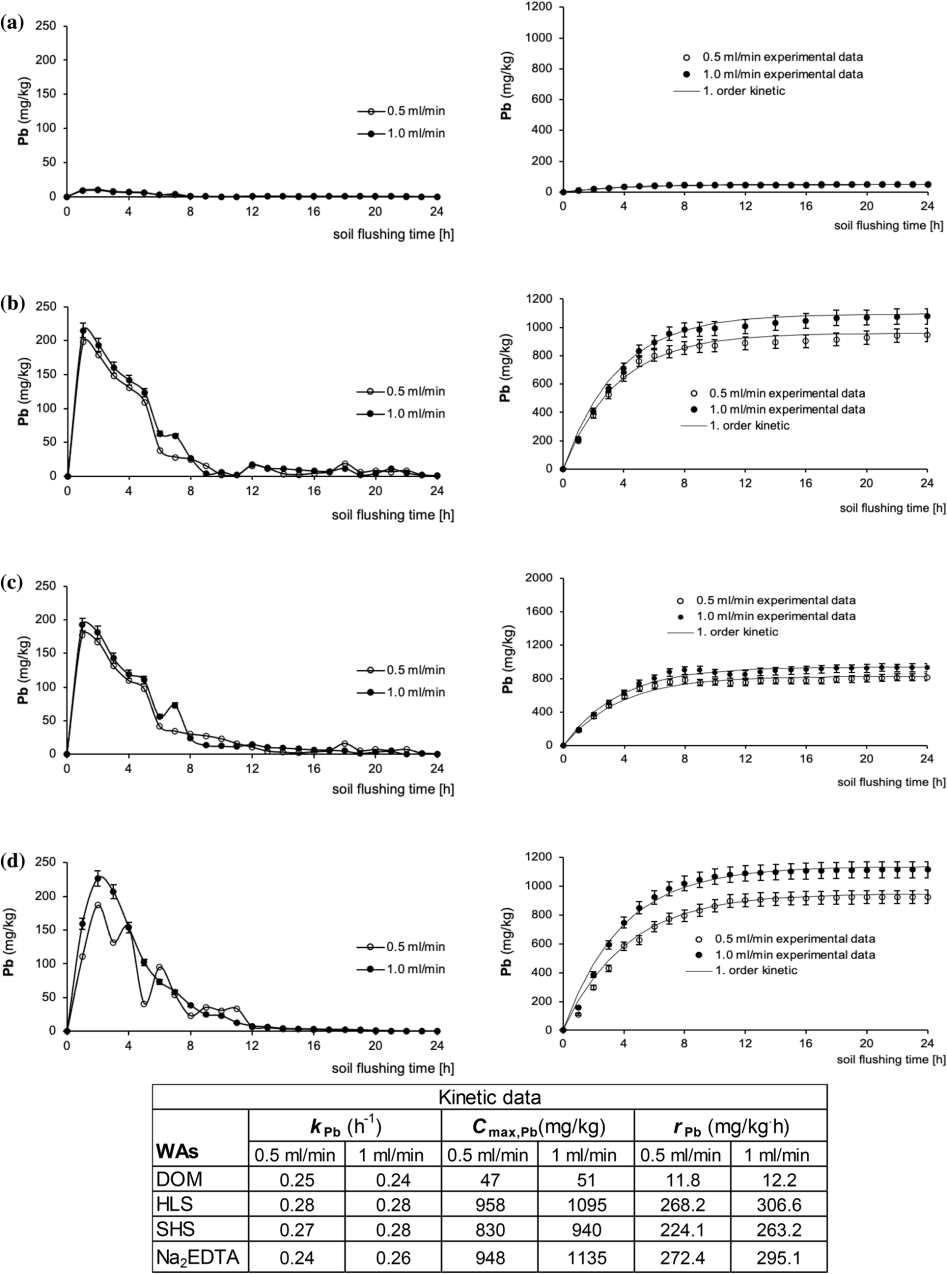
Pb mobilization curves and its cumulative removal during soil flushing at two flow rates with DOM (**a**), HLS (**b**), SHS (**c**), Na_2_EDTA (**d**) with kinetic constants. Data are expressed as mean values (n = 2) ± standard deviation error bars.

The analysis of TOC in the leachates from the column reactor revealed changes in the concentration of WAs available for HM removal (Fig. 4). In general, the changes in the concentrations of the SS_WAs tended to be similar to each other, but different from the changes in Na_2_EDTA concentration. At the beginning of soil flushing, the concentration of all SS_WAs was 5050 mg C/L (on average), whereas that of Na_2_EDTA was 638 mg C/L. The largest decrease in SS_WA concentrations took place between the first and fifth hours of flushing. Although sorption of SS_WAs was rapid, the drop in their concentration was not large: at a flow rate of 0.5 ml/min, 13%, 14% and 17% of DOM, HLS and SHS sorbed to soil, respectively; at 1.0 ml/min, 11%, 13% and 15% sorbed to soil. Although WA concentration can have an important influence on HM removal efficiency^29^, in the present study, the drop in SS_WA concentration was not large enough to disturb efficient HM removal, as shown by the fact that HM concentration removed from soil peaked after two hours of soil flushing (Figs. 4, 5, 6). Overall, the concentration of SS_WAs remained high enough to ensure HM removal. Moreover, as soil flushing continued, the concentration of SS_WAs rapidly increased, and at the end of the process, it was similar to the concentration at the beginning.

In contrast to what was observed with the SS_WAs, the TOC concentrations in the leachates from soil flushing with Na_2_EDTA were higher than the initial Na_2_EDTA concentration. This indicates that Na_2_EDTA dissolved native soil organic matter during soil flushing. TOC concentrations in Na_2_EDTA leachates were highest after the fifth hour of flushing. Tsang et al.^31^ have observed that little EDTA is adsorbed on soils and that it is transported as a non-sorbing solute during soil flushing. According to those authors, the leachate concentration of EDTA was equal to the influent concentration after 2–3 pore volumes of flushing. At a high concentration, EDTA can dissolve not only soil oxides and carbonates, but also organic matter. Those authors demonstrated that EDTA mobilized soil organic matter, which strongly interacts with Fe and Al oxides, and that this can facilitate HM removal from soil. In their study, as the EDTA concentration increased from 0.0001 M to 0.01 M, the concentration of dissolved organic carbon in the leachate from soil flushing increased from 12 to 1200 mg/L. In the present study, with Na_2_EDTA concentration of 0.005 M, the maximum concentration of dissolved organic matter (calculated as the difference between the TOC concentration in the leachate after the fifth hour of flushing and TOC in the Na_2_EDTA solution supplied to the reactor) was 91 mg/L at 0.5 ml/min and 98 mg/L at 1.0 ml/min.

### Kinetics of HM removal

It was found that, independently of the WA that was used, a large amount of Cu was already removed after the first hour of flushing: ranging from 997 mg/kg (with DOM) to 1118 mg/kg (with HLS). With DOM and Na_2_EDTA, but not the other WAs, the concentration of HM removed per hour was highest in the second hour of flushing (1174 mg/kg and 1097 mg/kg, respectively). The efficiency of Cu removal with DOM, HLS, and SHS gradually decreased from the 2^nd^-3^rd^ hour of flushing, and from the 8th hour of flushing, the amount of Cu removed per hour by these WAs was 130–150 mg/kg (Fig. 5). The curves of Cu mobilization with the different WAs were very similar. It should be emphasized that, in all cases, HM removal was more effective at the higher flow rate (1 ml/min). The largest differences between the amount of Cu flushed from the soil at each flow rate were about 150–200 mg/kg, and these differences were observed mainly during the first 2–3 h.

The results of cumulative amounts of Cu washed from soil (Fig. 5) clearly indicate that more flushing time is required for Cu removal with SS_WAs than with Na_2_EDTA. For example, with DOM (Fig. 5a, flow rate 1 ml/min), the concentration of Cu removed from soil after 8 h of flushing was 81% of the maximal concentration removed (obtained after 19 h of flushing). In contrast, with Na_2_EDTA (Fig. 5d, flow rate 1 ml/min), the concentration of the HM that was removed from soil after 8 h of flushing was 96% of the maximal concentration removed.

Based on the curves of cumulative Cu removal, kinetic constants were calculated (Fig. 5). The values of the kinetic constants for Cu removal (*k*_Cu_) with Na_2_EDTA differed considerably from the *k*_Cu_ values with the SS_WAs: with Na_2_EDTA, the respective *k*_Cu_ values were 0.36 and 0.37 h^−1^ at flow rates of 0.5 ml/min and 1 ml/min, 1.5–1.7-fold higher than these values with the SS_WAs (0.20–0.25 h^−1^). It should be strongly emphasized that, with all tested WAs, *k*_Cu_ values at the two flow rates did not differ considerably. However, it is also important that the flow rate influenced the maximum concentration of Cu removed from the soil (*C*_max,Cu_). With all of the next-generation WAs, *C*_max,Cu_ was 10–11% higher at a flow rate of 1 ml/min than at a rate of 0.5 ml/min, and with Na_2_EDTA, it was 20% higher at 1 ml/min.

The initial rate of Cu removal (*r*_Cu_) was highest when using Na_2_EDTA (1521.8 mg/kg·h at 0.5 ml/min, 1886.4 mg/kg·h at 1.0 ml/min), about 1.3–1.5-fold and 1.5–1.6-fold higher than when using the SS_WAs at flow rates of 0.5 ml/min and 1.0 ml/min, respectively. However, even though *k*_Cu_ and *r*_Cu_ were highest when using Na_2_EDTA, *C*_max,Cu_ was highest when using DOM. Therefore, although it requires more flushing time, DOM can remove more Cu from soil than Na_2_EDTA (at a flow rate 1 ml/min, DOM removed 73% of Cu; Na_2_EDTA removed 66% of Cu). Klik et al.^24^ found that DOM also removes Cu very effectively under batch conditions, using the same soil with the same Cu concentration as in this study. It was found that double soil washing with DOM removed 92% of Cu, whereas double washing with Na_2_EDTA removed 93%. Thus, DOM is very effective for removal of Cu from soil: under batch conditions, it removes as much Cu as Na_2_EDTA, and under column flushing conditions, it removes more Cu.

However, DOM is not an appropriate WA for removing Pb from soil. At both flow rates tested in the present study, DOM removed only ca. 50 mg/kg of Pb, corresponding to an efficiency of ca. 4%. Batch experiments with soil from a metallurgical area and spiked soil have also reported that DOM removes Pb with low effectiveness. For example, Kulikowska et al.^7^ reported that DOM removed Pb from soil from a metallurgical area with efficiency below 10%. Using the same soil as that used in the present study (with the same Pb concentration), Klik et al.^24^ found that Pb removal efficiency was 24%. Therefore, DOM should not be used to remove Pb from soils under batch conditions or via column flushing.

With the exception of DOM, the amount of Pb removed by other SS_WAs was highest in the first hour of removal, with 215 mg/kg removed by HLS and 192 mg/kg removed by SHS at flow rates of 1 ml/min. A peak in a mobilization curve for Pb removal with DOM was not noted (due to the very low efficiency of Pb removal). Pb removal with Na_2_EDTA was highest in the second hour of flushing (226 mg/kg; 1 ml/min). However, with Na_2_EDTA the difference in Pb removal between flow rates: at 0.5 ml/min Pb removal in the second hour was 187 mg/kg (1.2-fold lower than at 1 ml/min).

In contrast to the values of k_Cu_, the difference in the values of *k*_Pb_ for the SS_WAs and Na_2_EDTA were comparable (Fig. 6). Moreover, with all of the WAs, *k*_Pb_ values did not differ considerably between flow rates.

Although flow rate did not influence *k*_Pb_ values, it influenced the maximum concentration of Pb removed from soil (*C*_max,Pb_). With HLS, SHS and Na_2_EDTA, the respective values of *C*_max,Pb_ were by 14%, 13% and 20% higher at 1 ml/min than at 0.5 ml/min. Additionally, it should be emphasized that HLS, one of the next-generation WAs, removed Pb very effectively (1095 mg/kg; 77%); its effectiveness was only slightly lower than that of Na_2_EDTA (1135 mg/kg; efficiency 80%). The high *C*_max,Pb_ with HLS corresponded to the highest initial rate of Pb removal, *r*_Pb_ (306.6 mg/kg·h at 1.0 ml/min).

**Figure 7:**
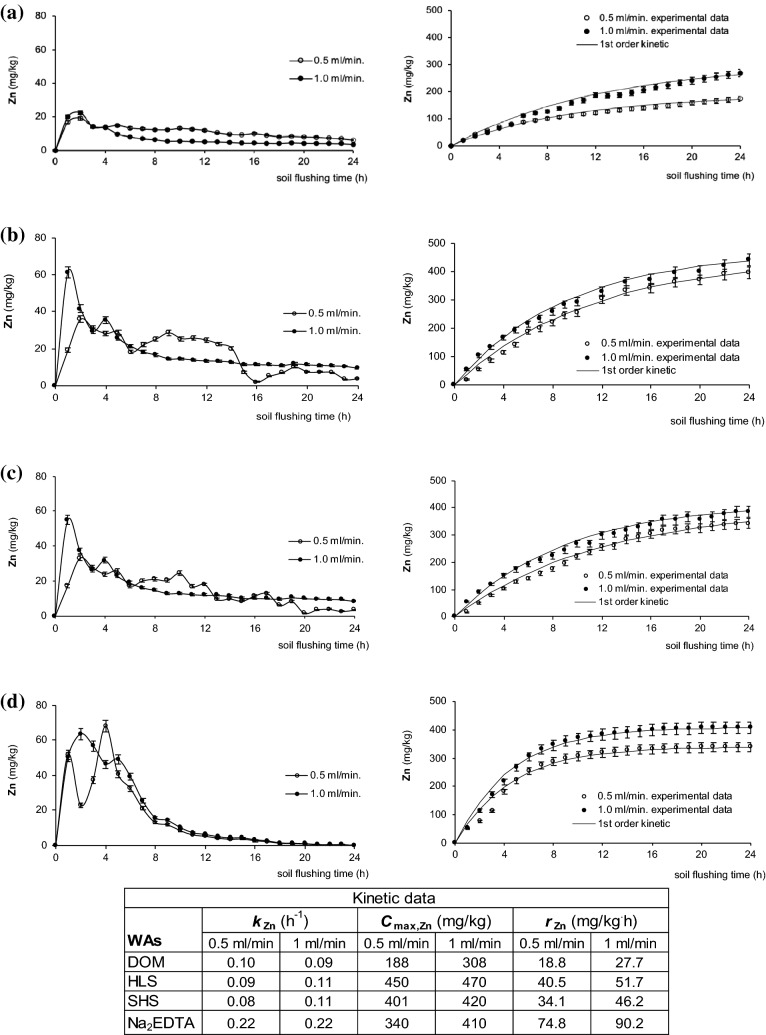
Zn mobilization curves and its cumulative removal during soil flushing at two flow rates with DOM (**a**), HLS (**b**), SHS (**c**), Na_2_EDTA (**d**) with kinetic constants. Data are expressed as mean values (n = 2) ± standard deviation error bars.

In contaminated soil, Zn was present in the lowest concentration (566 mg/kg). Zn leaching fluctuated more with HLS, SHS and Na_2_EDTA than with DOM (Fig. 7). Similar to what was observed with Cu and Pb, Zn removal was highest during the first few hours of soil flushing. Zn removal with WAs (except for DOM) fluctuated more with a flushing time at 0.5 ml/min than at 1.0 ml/min.

The cumulative amounts of Zn washed from soil are presented in Fig. 6. In the case of Zn, as in the case of Cu, a longer flushing time was required for Zn removal with the SS_WAs than with Na_2_EDTA. For example, with SHS (Fig. 6c, flow rate 1 ml/min), the concentration of Zn removed from soil after 8 h of flushing was 53% of *C*_max,Zn_. In contrast, with Na_2_EDTA (Fig. 6d, flow rate 1 ml/min), the concentration of the HM that was removed from soil after 8 h of flushing was 84% of *C*_max,Zn_ (although the overall process efficiency was similar, 74% for SHS and 72% for Na_2_EDTA). The differences in flushing time needed for effective Zn removal were reflected by the values of the kinetic constants, *k*_Zn_, which were about twofold higher with Na_2_EDTA than with the SS_WAs (0.22 h^−1^ at both flow rates vs. 0.09–0.11 h^−1^, respectively). With all tested WAs, *k*_Zn_ values did not differ significantly between flow rates. However, the flow rate influenced the maximum concentration of Zn removed from the soil (*C*_max,Zn_), but mainly with DOM and Na_2_EDTA.

The initial rate of Zn removal (*r*_Zn_) was highest with Na_2_EDTA (74.8 mg/kg·h at 0.5 ml/min, 94.2 mg/kg·h at 1.0 ml/min). However, even though *k*_Zn_ and *r*_Zn_ were highest with Na_2_EDTA, *C*_max,Zn_ was highest with HLS. Therefore, although it requires more flushing time, HLS can remove more Zn from soil than Na_2_EDTA (at a flow rate 1 ml/min, 83% Zn with HLS, 72% Zn with Na_2_EDTA).

This study shows that SS_WAs effectively remove Cu, Pb and Zn from soil. Although the rate of HM removal was higher with Na_2_EDTA, SS_WAs removed HMs with high efficiency within several hours. Hauser et al.^32^ obtained different results. In column experiment with EDDS as WA, flushing time lasted 5–8 weeks, depending on soil type and HM concentration: in 3 different soils, Cu concentrations ranged from 73 to 527 mg/kg; Zn concentrations, from 659 to 987 mg/kg; and those of Pb, from 60 to 732 mg/kg. They found that column flushing removed 18–26% of Cu, 20–64% of Zn and 18–91% of Pb. According to those authors, however, the main disadvantage of column leaching was the long flushing time, which caused substantial biodegradation and dissolution of iron oxides leading to the formation of Fe(III)EDDS and the biodegradation of EDDS. These processes reduced the flushing efficiency because less EDDS was available for complexation.

Juwarkar et al.^17^ used di-rhamnolipid biosurfactant for removal of Pb (905.4 mg/kg) and Cd (435.4 mg/kg) in a column experiment. Cumulative removal of Pb and Cd after 36 h flushing, totaled 92% and 88%, respectively. Although cumulative Pb removal with HLS in the present study (77%) was lower than the amount removed with di-rhamnolipid biosurfactant^17^, it should be taken into account that the amount Pb in the contaminated soil in the present study (1414 mg/kg) was almost 60% higher than that in Juvarkar et al.^17^. Moreover, the amount of Pb removed in the present study totaled 1100 mg/kg, whereas the amount in their study equaled 800 mg/kg. Additionally, the time for Pb removal was ca. twofold shorter with HLS than with di-rhamnolipid (18 h versus 36 h). It should be emphasized, however, that the course of Pb removal differed between these two studies. Juvarkar et al.^17^ found that, at the time of the first leachate sample, 8% of Cd had been removed from the soil, and at 24 h, 30 h and 36 h, 20% of the HM had been removed. Pb removal was 4% at the time leachate was first sampled, followed by 8% at 6 h, 12% at 12 h and 20% at 24 h. Later, Pb removal remained constant at 22% at 30 and 36 h. In the study presented here, in contrast, the hourly amount of Pb removed by the SS_WAs was highest in the first 2 h of removal, with 408 mg/kg removed by HLS during this time (37% of *C*_max,Pb_), and 982 mg/kg of Pb had been removed after 8 h (90% of *C*_max,Pb_).

### HM mobilization from chemical fractions as a factor determining soil flushing efficiency

**Figure 8:**
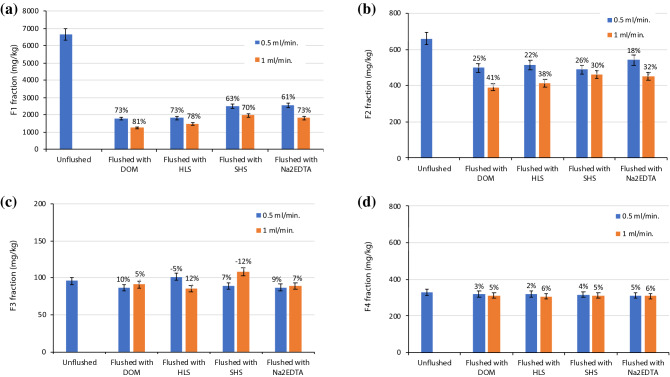
Changes in Cu distribution in unflushed soil and soil flushed with DOM, HLS, SHS and Na_2_EDTA at two flow rates. The values above the bars mean the removal efficiency of Cu from individual fractions: (**a**) F1 fraction, (**b**) F2 fraction, (**c**) F3 fraction, (**d**) F4 fraction. Data are expressed as mean values (n = 2) ± standard deviation error bars.

**Figure 9:**
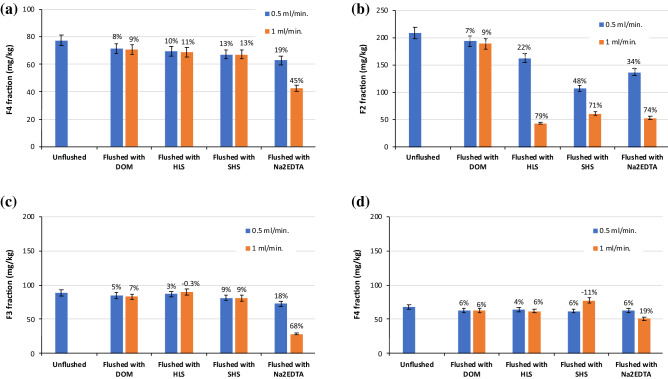
Changes in Pb distribution in unflushed soil and soil flushed with DOM, HLS, SHS and Na_2_EDTA at two flow rates. The values above the bars mean the removal efficiency of Pb from individual fractions: (**a**) F1 fraction, (**b**) F2 fraction, (**c**) F3 fraction, (**d**) F4 fraction. Data are expressed as mean values (n = 2) ± standard deviation error bars.

**Figure 10:**
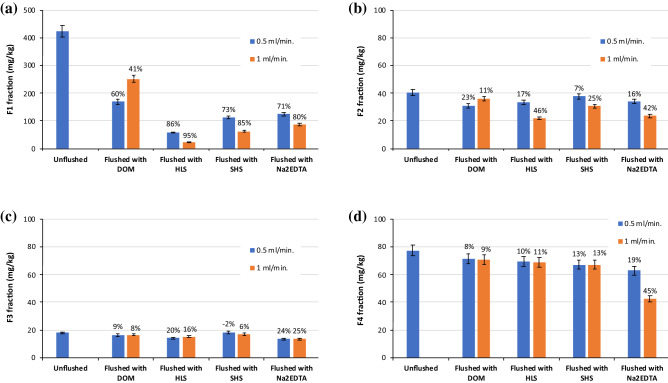
Changes in Zn distribution in unflushed soil and soil flushed with DOM, HLS, SHS and Na_2_EDTA at two flow rates. The values above the bars mean the removal efficiency of Zn from individual fractions: (**a**) F1 fraction, (**b**) F2 fraction, (**c**) F3 fraction, (**d)** F4 fraction. Data are expressed as mean values (n = 2) ± standard deviation error bars.

The changes in Cu, Pb and Zn distribution in unflushed and flushed soil are presented in Figs. 8, 9 and 10. In contaminated soil, the content of the HMs in the fractions was as follows: Cu, F1 >  > F2 > F4 > F3; Pb, F1 >  > F2 > F3 > F4; and Zn, F1 >  > F4 > F2 > F3. Due to their high mobility, Cu, Pb and Zn were susceptible to removal from soil via flushing. Indeed, the share of each HM in the exchangeable and acid soluble (F1) fraction ranged from 74 to 86% of the total concentration. In contrast, the shares of the HMs in the potentially mobile fraction, F2, were markedly lower: 7.3% of Zn, 8.5% of Cu and 14.8% of Pb. The share of each HM in the oxidizable (F3) fraction was low (1.2–6.4%), which reflected soil properties affecting sorption capacity, i.e., low organic matter content and low cation exchange capacity^19^. Similarly, the shares in the residual (F4) fraction ranged from 4.2% (Cu) to 13.8% (Zn). In general, the higher the content of a HM in the F3 and F4 fractions, the lower the efficiency of removal of the HM^33^.

The analysis of HM distribution indicates that, during soil flushing, HMs were mainly removed from F1 and F2 fractions. In addition, the higher WA flow rate facilitated HM removal from these fractions. The low removal efficiency of Cu from stable F3 and F4 fractions resulted from high share of HM in the most mobile F1 and F2 fractions and their preferable removal with WAs.

At a flow rate of 0.1 ml/min, DOM and HLS decreased Cu content in the F1 fraction from 6655 mg/kg to 1236 and 1486 mg/kg, corresponding to 81 and 78% efficiency, respectively. This was more efficient than Cu removal with SHS and Na_2_EDTA. DOM and HLS also removed Cu efficiently from the F2 fraction. The relative amounts of Cu removed from the F1 and F2 fractions by the WAs corresponded well with the total amounts removed, which were higher with DOM and HLS than with SHS and Na_2_EDTA.

The effect of soil flushing at the two flow rates on Cu removal from the oxidizable fraction (F3) was clear. With HLS at a flow rate of 0.5 ml/min, and with SHS at 1.0 ml/min, the content of Cu in the F3 fraction increased from 97 mg/kg to 101 and 107 mg/kg, respectively. This slight increase in Cu content could be due to sorption of WAs in the soil, and redistribution and incorporation of previously mobilized HMs into the F3 fraction.

Although the soil had been spiked, the Cu in the residual fraction (F4) was relatively stable. With all tested WAs, the efficiency of Cu removal from the F4 fraction was only 2–6%. HMs in the residual fraction are usually expected to be very stable and generally cannot be removed or redistributed into other fractions^34^. In contrast, those in the exchangeable, carbonate and oxide bound fractions are easily extracted from soil under washing conditions using organic chelators^33,35^. Race et al.^36^ observed that flushing with EDDS mainly removed Cu and Zn from the acid soluble fraction, which corresponded to rapid HM mobilization.

Similarly, in our study, the HM concentrations in the leachate from the column reactor were highest during the first 1–3 h of soil flushing. Efficient removal of HMs from the exchangeable and acid soluble fraction can be caused by ion exchange and metal dissolution from carbonates. Ke et al.^37^ have shown that, during column leaching of HMs (including Cu, Pb and Zn) from industrial soils using citric acid as an organic chelator, the HMs were mainly removed from the exchangeable and carbonate fractions (the F1 fraction in our study) and from the reducible fraction. The main reason for HM removal was solubility of exchangeable fraction with citric acid, while the principal cause of HM removal from the reducible and oxidizable fractions was chelation with citric acid.

At the flow rates tested in this study, the overall efficiency of Pb removal with DOM was poor (Fig. 8), as was the efficiency of its removal from each fraction with this WA (4 to 9%). After application of HLS and Na_2_EDTA at 0.5 ml/min, the content of Pb in the F1 fraction decreased by 80–84%. An increasing in flow rate was not significant for further Pb removal from the F1 fraction. On contrary, flow rate of 1.0 ml/min was beneficial for decreasing Pb content in the F2 fraction, i.e. from 162 to 44 mg/kg for HLS and from 137 to 53 mg/kg for Na_2_EDTA. On this basis, the efficiency of Pb removal from the F2 fraction at 1.0 ml/min was higher by 57% and by 40%, respectively compared to Pb removal at 0.5 ml/min. In contrast to Na_2_EDTA, the SS_WAs had little effect on Pb removal from F3 and F4 fractions. Complexation of Pb with soil organic matter limits its migration, this may explain poor decrease in oxidizable Pb after flushing with SS_WAs, which partially sorbed to soil^38^. Na_2_EDTA is considered to be the most effective synthetic chelator to remove Pb from soil due to its strong chelating ability for Pb^39,40^. Chelating agents are capable of partly dissolving soil matrix constituents including (hydr)oxides of major HMs as well as organic matter^18^.

The ability of WAs to remove Zn from the F1 fraction at both flow rates was the highest for HLS, lower but comparable for SHS and Na_2_EDTA, and the lowest for DOM. Soil flushing with HLS at 1.0 ml/min nearly completely removed Zn from the most mobile fraction. Based on HM changes in the content of F1 fraction, DOM affinity towards to HMs decreased in order: Cu > Zn > Pb. The lowest Zn concentration in the F2 fraction was observed after soil flushing with HLS and Na_2_EDTA. These two WAs were also the most efficient in Zn removal from the F3 fraction. The SS_WAs and Na_2_EDTA visibly differed in Zn removal from the F4 fraction, 8–13% versus 19–45%, respectively.

## Conclusions

Soil flushing in a column with SS_WAs can be considered a method for remediation of Cu, Pb and Zn contaminated soil. At both flow rates and for all WAs used, HMs were removed mainly from the F1 and F2 fractions. With all the WAs at both flow rates, stable flow velocity was maintained for approximately 8 h at 1.0 ml/min and 16 h at 0.5 ml/min. With longer flushing times (up to 24 h) and larger pore volumes, hydraulic conditions became unstable.

Although flow rate did not influence *k,* it influenced the maximum concentration of each individual metal removed: with almost all tested metals and WAs, *C*_max_ values were higher at 1 ml/min than at 0.5 ml/min. At both flow rates, *k* values for Cu and Zn removal, but not Pb removal, were ca. twofold higher with Na_2_EDTA than with SS_WAs.

Of the tested SS_WAs, DOM removed Cu most effectively (73%), and HLS removed Zn and Pb most effectively (83% and 77%, respectively). Thus, compared to removal of the respective metals with Na_2_EDTA (63%, 72% and 80%), DOM removes Cu more efficiently, HLS removes Zn more efficiently, and HLS removes Pb only slightly less efficiently.

The large decreases in HM concentrations (*C*_max_) that were observed indicate that soil flushing with SS_WAs can be successfully applied for moderately contaminated soil. For effective Cu removal, DOM is the best alternative; for Pb and Zn removal, HLS is the best one.
